# Effect of *Saccharomyces cerevisiae* CICC 32883 Fermentation on the Structural Features and Antioxidant Protection Effect of Chinese Yam Polysaccharide

**DOI:** 10.3390/foods14040564

**Published:** 2025-02-08

**Authors:** Zichao Wang, Yi Zheng, Ziru Lai, Zhihao Kong, Xilei Hu, Peiyao Zhang, Yahui Yang, Na Li

**Affiliations:** 1 School of Biological Engineering, Henan University of Technology, Zhengzhou 450001, China; 2 School of International Education, Henan University of Technology, Zhengzhou 450001, China; 3 Editorial Department of Journal of Henan University of Technology, Zhengzhou 450001, China; 4 Henan Institute of Medical and Pharmaceutical Sciences, Zhengzhou University, Zhengzhou 450001, China

**Keywords:** Chinese yam polysaccharide, *Saccharomyces cerevisiae* CICC 32883, fermentation, structural features, antioxidant activity

## Abstract

In this study, Chinese yam polysaccharide (CYP) was fermented by *Saccharomyces cerevisiae* CICC 32883, and its structural features and antioxidant activities before and after fermentation were analyzed. *S. cerevisiae* CICC 32883-fermented CYP (CYP-SC) had higher carbohydrate content and lower protein content than the nonfermented CYP (CYP-NF). The monosaccharide composition of CYP-SC was unaffected, but the proportion was changed. Compared with CYP-NF’s molecular weight and polydispersity of 124.774 kDa and 6.58, respectively, those of CYP-SC were reduced to 20.384 kDa and 3.379. Antioxidant results showed that CYP-SC had better effects than CYP-NF in scavenging DPPH, ABTS, hydroxyl, and superoxide radicals. Moreover, CYP-SC showed better activities in enhancing oxidation capacity and protecting HepG2 cells than CYP-NF. Furthermore, the effects of CYP-SC on alleviating and repairing H_2_O_2_-damaged HepG2 cells are superior to those of CYP-NF. This work offers a green and efficient method for enhancing the antioxidant activity of dietary plant polysaccharides.

## 1. Introduction

Excessive reactive oxygen species (ROS) attack normal cells and tissues in the body, resulting in a loss of elasticity and dullness of the skin in light cases and wrinkles and the formation of color spots in others [1,2]. ROS can also cause mutations in nucleic acids, which primarily cause human aging and diseases [3,4,5]. Apart from reasonable diet and exercise, the consumption of foods containing antioxidant compounds, particularly plant-derived bioactive compounds, is a good choice to remove ROS [6,7]. Rudrapal et al. [8] suggested that plant polyphenols have antioxidant effects and can be used to manage diseases (including hypertension, allergies, aging, and chronic diseases). Plant essential oils can not only be used for food preservation but also have good antioxidant properties and disease prevention effects [9,10]. Wu et al. [11] found that phytochemicals have antioxidant and anti-inflammatory effects and can be used as ingredients in functional foods. Chaudhary et al. [12] suggested that plant alkaloids have antioxidant effects and can be used to treat diabetes. Cesar et al. [13] found that plant peptides can slow down oxidative processes in the body and improve human health. Bai et al. [14] demonstrated that plant polysaccharides with antioxidant activity can be potentially used for biomedical and medicinal applications. In our previous studies, we verified that *Artemisia argyi* flavonoids have excellent antioxidant activity and potential applications as food preservatives and ingredients [15,16]. Therefore, developing plant-derived antioxidant compounds is important to body health.

As a biopolymer, the polysaccharide is connected by glycosidic bonds via more than 10 monosaccharides; it possesses not only antibacterial, anti-inflammatory, hypoglycemic, hypolipidemic, anti-tumor, immunomodulatory, prebiotic, and other bioactivities [17,18,19,20,21,22], but also antioxidant activities [19,20,23,24], particularly those from dietary plants. Chinese yam is a rhizome of *Dioscorea* that is both a medicine and a food plant, which not only contains rich nutrients such as starch, amino acids, proteins, vitamins, and mineral elements, but also includes polysaccharides, polyphenols, allantoin, diosgenin, and other bioactive products [25]. Its polysaccharides possess numerous bioactivities, including antioxidant activity [26]. Zhou et al. [27] found that Chinese yam bulbil polysaccharides have antioxidant and antifatigue effects. Shao et al. [28] suggested that the Chinese yam peel polysaccharides have antioxidant, immunomodulatory, and hypoglycemic effects. However, relatively low antioxidant activity restricts the application of yam polysaccharides. Apart from physical, chemical, and enzymatic strategies, organic acids and/or enzymes released by microorganisms can destroy plant cells and increase polysaccharide extractability [29]. They can also modify the polysaccharide structure and enhance its antioxidant activity [30]. Hence, microbial fermentation may be a good choice to enhance the antioxidant activity of yam polysaccharide with the advantages of environmental friendliness, low reaction temperature, and energy saving.

*Saccharomyces* was one of the earliest microorganisms used by human beings. It has been widely applied in fermented food and brewing production. In recent years, many studies have suggested that *Saccharomyces* fermentation can improve the antioxidant activity of plant polysaccharides. Shao et al. [31] verified that *S. boulardii* fermentation improves the antioxidant, radioprotective, and digestive effects of Chinese yam polysaccharides (CYPs). Wang et al. [32] suggested that the antioxidant and antiaging effects of *Lycium barbarum* polysaccharide are enhanced via rice wine yeast fermentation. Chen et al. [33] found that *S. cerevisiae* fermentation increases the antioxidant and anti-inflammatory effects of *Dendrobium officinale* polysaccharide. Pei et al. [34] indicated that *S. cerevisiae*-fermented blue honeysuckle polysaccharide has high antioxidant and *α*-amylase inhibitory activities and TG binding capacity. Additionally, the combined fermentation by *Saccharomyces* and other bacteria is utilized to enhance the antioxidant activity of plant polysaccharides [35,36]. Therefore, *Saccharomyces* fermentation can offer a green and efficient method for enhancing the antioxidant activity of CYP.

Herein, *S. cerevisiae* CICC 32883 was used to ferment and modify Chinese yam polysaccharides (CYPs). The chemical composition of CYPs before and after fermentation was detected using spectrophotometric colorimetry. Then, monosaccharides and molecular weights were determined via high-performance anion-exchange chromatography and high-performance size exclusion chromatography. Functional groups were characterized with Fourier transform infrared (FT-IR) spectroscopy. Moreover, the antioxidant activities of CYPs before and after fermentation were analyzed with in vitro and H_2_O_2_-damaged HepG2 cell models.

## 2. Materials and Methods

### 2.1. Materials and Microorganisms

Chinese yam and *S. cerevisiae* CICC 32883 were purchased from a local supermarket in Zhengzhou (Zhengzhou, China) and the China Center of Industrial Culture Collection (Beijing, China), respectively. Yeast mold (YM) broth medium (Cat: LA5171) was purchased from Beijing Solarbio Science & Technology Co., Ltd. (Beijing, China). Ethanol, trichloromethane, trifluoroacetic acid (TFA), n-butanol, H_2_O_2_, dialysis bag, and activated carbon were purchased from Beijing Solarbio Science & Technology Co., Ltd. (Beijing, China). Glucose, bovine serum albumin, gallic acid, potassium bromide, salicylic acid, ABTS, and other reagents were purchased from Beijing Solarbio Science & Technology Co., Ltd. (Beijing, China). Standard monosaccharides and dextran were purchased from Sigma-Aldrich (Shanghai, China). The enzyme-linked immunosorbent assay (ELISA) kits used for determining superoxide dismutase (SOD), catalase (CAT), glutathione peroxidase (GSH-Px), and malondialdehyde (MDA) were purchased from Beyotime Biotechnology (Shanghai, China).

### 2.2. Extraction of CYPs

Chinese yams were peeled and cut into small pieces. Then, they were crushed into a paste by using a grinder. One part of the pasted Chinese yam was added to nine volumes (*w*/*v*) of deionized water and stirred magnetically under room temperature for 8 h. Afterward, Chinese yam solution was collected and centrifuged at 8000× *g* for 10 min to remove insoluble matters. The activated carbon was added to the supernatant with a ratio of 1 g/100 mL and was decolorized at 150 r/min overnight at room temperature. Then, it was centrifuged at 8000× *g* for 10 min to collect the supernatant. The supernatant was concentrated to one-fifth under 60 °C and 0.1 MPa via a vacuum pump. Then, three volumes of Sevag solution (trichloromethane: n-butyl alcohol = 3:1) were added, shaken vigorously, and centrifuged at 10,000× *g* for 10 min to collect the supernatant. Afterward, five volumes of alcohol were added into the supernatant and placed at 4 °C overnight to precipitate crude CYP. Subsequently, the precipitated CYP was redissolved in water and rotary-evaporated at 60 °C and 0.1 MPa. The CYP concentrate was desalted by dialysis (molecular weight cut-off was 10.0 kDa) in deionized water for 48 h by changing the water every 4 h. Finally, the dialytic solution of nonfermented CYP (CYP-NF) was collected and lyophilized.

Another part of the pasted Chinese yam was added to nine volumes (*w*/*v*) of deionized water, peptone (1.0 g/L), yeast extract (1.0 g/L), MgSO_4_ (1.0 g/L), KH_2_PO_4_ (1.0 g/L), K_2_HPO_4_ (1.0 g/L), and 200 mL fermentation liquid contained in a 500 mL flask. Then, it was sterilized at 80 °C for 60 min. Then, the *S. cerevisiae* CICC 32883 that grew to a logarithmic phase in the YM broth medium was inoculated with 10% volume (*v*/*v*) and cultured at 30 °C for 168 h, and the shaking speed was set as 180 r/min following the report of Shao et al. [31] with slight modifications. After fermentation, the fermentation broth was collected and filtered with eight layers of gauze to remove large sediments. Then, it was centrifuged at 10,000× *g* for 10 min to collect the supernatant. Then, the extraction process of *S. cerevisiae* CICC 32883-fermented CYP (CYP-SC) was the same as that of CYP-NF.

### 2.3. Basic Composition Analysis

The carbohydrate content in CYP-NF and CYP-SC was detected using the anthrone–sulfonic acid colorimetric method with glucose as the standard; the polyphenol content was determined using the Folin–Ciocalteu method with gallic acid as the standard; and the protein content was measured using the Coomassie Brilliant Blue method with bovine serum albumin as the standard [37].

### 2.4. Monosaccharide Detection

CYP-NF (or CYP-SC) was dissolved in 2 M TFA and hydrolyzed at 120 °C for 2 h. Then, hydrolysate was washed three times with methanol and evaporated to dryness to remove TFA. Finally, the hydrolyzed material was transferred to a 25 mL volumetric flask, diluted to 25 mL by using deionized water, and subjected to a Dionex ICS5000 system (Dionex, Sunnyvale, CA, USA) equipped with a CarboPac PA20 column (ID: 3 mm × 150 mm). The mobile phase was deionized water (A), 0.25 M NaOH (B), and 1 M NaAc (C) and eluted as follows (A%, B%, and C%): 0 min: 99.2, 0.8, and 0; 30 min: 99.2, 0.8, and 0; 40 min: 79.2, 0.8, and 20; 40.1 min: 20, 80, and 0; 60 min: 99.2, 0.8, and 0. The flow rate was 0.45 mL/min, and the injection volume was 25 µL. The column temperature was 30 °C and the detection instruments were a pulsed ampere detector, Au electrode, and Ag/AgCl reference electrode. Fucose, rhamnose, arabinose, glucosamine, galactose, glucose, xylose, mannose, fructose, ribose, galacturonic acid, mannuronic acid, guluronic acid, and glucuronic acid were used as the standard monosaccharides in this work.

### 2.5. Molecular Weight Detection

CYP-NF (or CYP-SC) was dissolved in distilled water to a concentration of 2 mg/mL. The detection system consisted of a Waters 2695 HPLC system equipped with multiple detectors: a refractive index (RI) detector and a UV detector for concentration determination, a multiple-angle laser light scattering detector (MALLS, DAWNHELEOS, Wyatt Technology, Goleta, CA, USA) for direct molecular weight determination, and a differential pressure viscometer for viscosity determination. TSK PWXL 6000 and 3000 gel filtration columns were eluted with PB buffer (0.15 M NaNO_3_ and 0.05 M NaH_2_PO_4_, pH = 7) at a flow rate of 0.5 mL/min. The laser photometer was calibrated with ultrapure toluene. Normalization was conducted with a bovine serum albumin globular protein (Mw = 66.7 kDa, Rg = 2.9 nm). A value of 0.146 mL/g was used as the RI increment (dn/dc) for molecular weight calculation. Astra software (Version 6.1.1) was utilized for data acquisition and analysis. The column temperature and the RI detector temperature were maintained at 35 °C.

### 2.6. FT-IR Spectroscopy Analysis

Approximately 1 mg freeze-dried CYP-NF (or CYP-SC) and 100 mg potassium bromide were added into the mortar, ground thoroughly, and pressed to the tablet for detection by using a Nexus 470 FT-IR spectrophotometer (Nicolet, Edgar, WI, USA). The sample was scanned via infrared spectroscopy from 500 cm^−1^ to 4000 cm^−1^.

### 2.7. In Vitro Antioxidant Activity of CYPs

CYP-NF and CYP-SC were dissolved in deionized water in concentrations of 0.5, 1.0, 1.5, 2.0, and 2.5 mg/mL. Then, the CYP-NF and CYP-SC solutions were filtrated through a 0.22 μm aqueous membrane; then, the antioxidant activities of CYP-NF (or CYP-SC) against DPPH, ABTS, hydroxyl, and superoxide radicals were detected according to methods reported previously [19].

#### 2.7.1. DPPH Radical Scavenging Activity

Equal amounts of CYP-NF (or CYP-SC) solution were mixed with an ethanol-dissolved 0.1 mM DPPH solution and incubated in the dark for 40 min at room temperature. The measured value of the mixture at the absorbance of 517 nm was set as A_i_. Instead of 0.1 mM ethanol, DPPH was used as the control group, and its absorbance was set as A_j_. For the blank group, deionized water replaced the sample solution, and its absorbance was set as A_0_. DPPH radical scavenging activity% = (A_0_ − A_i_ + A_j_)/A_0_ × 100%.

#### 2.7.2. ABTS Radical Scavenging Activity

Equal amounts of 7.4 mM ABTS solution and potassium 2.6 mM persulfate were mixed and incubated in the dark overnight at room temperature. Then, 7.4 mM ABTS was diluted with a neutral phosphate buffer to obtain its working solution until the absorbance at 734 nm was 0.7. After the ABTS working solution was mixed with the CYP-NF (or CYP-SC) solution in a 4:1 ratio and left to stand for 6 min, the absorbance measured at 734 nm was set as A_i_. Then, a 95% ethanol-replaced sample solution was used as the blank group, and its absorbance was set as A_0_. ABTS radical scavenging activity% = (A_0_ − A_i_)/A_0_ × 100%.

#### 2.7.3. Hydroxyl Radical Scavenging Activity

Equal parts of ferrous sulfate (6 mM), H_2_O_2_ (6 mM), CYP-NF (or CYP-SC) solution, and salicylic acid (6 mM) were mixed and stood for 30 min at 37 °C. The mixture at the absorbance of 510 nm was set as A_i_. The H_2_O_2_ replaced by deionized water was used as the control group, and its absorbance was set as A_j_. The sample solution replaced by deionized water was used as the blank group, and its absorbance was set as A_0_. Hydroxyl radical scavenging activity% = (A_0_ − A_i_ + A_j_)/A_0_ × 100%.

#### 2.7.4. Superoxide Anion Scavenging Activity

The CYP-NF (or CYP-SC) solution was briefly mixed with equal parts of nitro blue tetrazolium (0.3 mM), reduced nicotinamide adenine dinucleotide (0.936 mM), and methyl sulfate phenol (0.12 mM). The mixture was incubated for 5 min at 25 °C, and the absorbance at 560 nm was determined as A_i_. The deionized water replaced by the sample solution was used as the blank group, and its absorbance was set as A_0_. Superoxide anion scavenging activity% = (A_0_ − A_i_)/A_0_ × 100%.

### 2.8. Construction of H_2_O_2_-Damaged HepG2 Cell Model

The H_2_O_2_-damaged HepG2 cell model was established according to the method reported previously with slight modifications [38]. HepG2 cells were cultured in Dulbecco’s Modified Eagle Medium (DMEM) containing 10% (*v*/*v*) fetal bovine serum, 100 µg/mL streptomycin, and 100 µg/mL penicillin at 5% (*v*/*v*) CO_2_ and 37 °C. HepG2 cells were continuously digested using 0.25% (*w*/*v*) trypsin ethylene diamine tetraacetic acid solution during culture. Afterward, the HepG2 cells in the logarithmic phase at a concentration of 2 × 10^5^ cells/mL were transferred to 96-well plates. Then, 100 μL H_2_O_2_ with different concentrations (0.03, 0.06, 0.09, 0.12, and 0.15 mmol/L) was added separately and incubated for another 6 h. The viability of HepG2 cells was measured using a CCK-8 kit and H_2_O_2_ concentration, at which approximately 50% HepG2 cell death was selected for the following investigations.

### 2.9. Antioxidant Activity of CYPs on the HepG2 Cell Model

#### 2.9.1. CYPs on the Oxidation Capacity and H_2_O_2_-Damaged Prevention of HepG2 Cells

CYP-NF and CYP-SC were separately dissolved into DMEM to concentrations of 5.0, 2.5, 1.25, 0.625, and 0.3125 mg/mL and were filtrated through 0.22 μm aqueous membrane. The HepG2 cells cultured in the logarithmic phase were transferred into 24-well plates with a concentration of 2 × 10^5^ cells/mL, and the culturing medium was discarded. Then, 100 μL different concentrations (5.0, 2.5, 1.25, 0.625, and 0.3125 mg/mL) of CYP-NF (or CYP-SC) samples were added separately into 24-well plates and incubated for another 24 h. Afterward, one part of HepG2 cells was collected and washed twice with phosphate-buffered solution (pH = 7.2–7.4); another part of HepG2 cells was treated with 100 μL 0.09 mmol/L H_2_O_2_ for 6 h, collected, and washed with phosphate-buffered solution. Finally, two parts of HepG2 cells were lysed by lyase and centrifugated at 8000× *g* for 10 min to obtain supernatants. Antioxidant enzymes (SOD, CAT, GSH-Px, and MDA) in different HepG2 cells were detected using ELISA kits according to the manufacturer’s protocols.

#### 2.9.2. CYPs on H_2_O_2_-Damaged Alleviation and Repair of HepG2 Cells

The HepG2 cells in the logarithmic phase were transferred into 24-well plates and adjusted to a concentration of 2 × 10^5^ cells/mL. Then, the culturing medium was discarded. One part of HepG2 cells was added to 50 μL different concentrations (10.0, 5.0, 2.5, 1.25, and 0.625 mg/mL) of CYP-NF (or CYP-SC) and 50 μL 0.18 mmol/L H_2_O_2_ and was cultured for 6 h; another part of HepG2 cells was treated by 100 μL 0.09 mmol/L H_2_O_2_ for 6 h. Then, the culturing medium was discarded, and 100 μL different concentrations (5.0, 2.5, 1.25, 0.625, and 0.3125 mg/mL) of CYP-NF (or CYP-SC) were separately added and incubated for another 24 h. Finally, two parts of HepG2 cells were collected, processed, and detected in the same way as those in Section 2.9.1.

### 2.10. Statistical Analysis

All data were expressed as mean ± SD after three repetitions. The data were analyzed via the analysis of variance using Origin software (Origin Pro 8.5).

## 3. Results and Discussion

### 3.1. Chemical Composition Analysis

As shown in Table 1, the carbohydrate content in CYP-NF was 71.03% ± 2.75%, which increased to 84.86% ± 2.92% in CYP-SC after being fermented by *S. cerevisiae* CICC 32883. Compared with CYP-NF’s protein and polyphenol contents of 8.24% ± 0.19% and 0.26% ± 0.07%, respectively, those of in CYP-SC decreased to 7.12% ± 0.19% and 0.13% ± 0.04%. On the one hand, yam species and extraction sites could affect the chemical composition of CYPs. Ju et al. [39] found that the CYP extracted from the *D. opposite* rhizome contains 63.2% total sugar and 15.2% protein. However, Li et al. [40] reported that the polysaccharide fraction of the DOP-2 extracted from the *D. opposite* rhizome contains 70.40% ± 1.69% total sugar and 1.42% ± 0.13% protein. Zhou et al. [27] found that the total carbohydrate contents were 89.18% ± 1.83% and 63.55% ± 1.21% in the Chinese yam bulbil polysaccharide fractions PYB-1 and PYB-2, respectively. Shao et al. [28] found that the total sugar and protein contents in the polysaccharide fraction CYPP-1 extracted from the Chinese yam peel were 77.42% ± 1.68% and 0.74% ± 0.02%, respectively. On the other hand, the extraction and purification methods affect the chemical composition of CYPs. Liu et al. [41] found that the polysaccharide contents of fractions CYP1, CYP2, and CYP3 using cellulase-assisted extraction methods were 88.6%, 87.4%, and 89.1%, respectively. Shao et al. [28] found that the total sugar content increases from 38.92% ± 0.77% to 77.42% ± 1.68% in the polysaccharide fraction of the CYPP-1 extracted from the Chinese yam peel via purification. Although many scholars did not detect polyphenols in CYPs, Shao et al. [28] found that the fraction of the CYPP-1 extracted from the Chinese yam peel contains 0.22% ± 0.01% total polyphenols. This finding is similar to the result in the present work. Furthermore, organic acids and/or enzymes released by *S. cerevisiae* CICC 32883 can destroy the connections between polysaccharides and proteins and polyphenols [42,43], thereby considerably reducing the protein and polyphenol contents in the extracted CYP-SC compared with those in CYP-NF (Table 1). The results of the present work may provide good references for enhancing the purity of plant-based bioactive compounds or promoting bioactive compound release from plants via microbial fermentation. Unfortunately, because the effect of *S. cerevisiae* CICC 32883 fermentation on yam polysaccharides was only preliminarily studied in this work, the fermentation kinetics and optimized extraction conditions were not thoroughly investigated.

### 3.2. Monosaccharide Analysis

As shown in Table 1, the monosaccharide composition and proportion in CYP-NF were rhamnose, arabinose, galactose, glucose, and mannose in a molar ratio of 0.493:0.6695:0.9738:0.7655:12.4365. CYP-SC also comprised rhamnose, arabinose, galactose, glucose, and mannose. However, the molar ratio changed to 0.1543:0.1635:0.5759:0.5286:3.2247. Similar to the chemical composition results, the monosaccharide composition and proportion of CYPs were also affected by yam species and extraction methods. Ju et al. [39] found that the CYP extracted from *D. opposite* rhizome is composed of mannose, glucose, galactose, and glucuronic acid in a molar ratio of 0.5:1.2:0.3:0.3. However, Li et al. [40] reported that the main monosaccharides in the CYP extracted from the *D. opposite* rhizome are glucose, galactose, and arabinose with a molar ratio of 26.12:53.45:20.43. Shao et al. [28] found that the Chinese yam peel polysaccharide fraction CYPP-1 is composed of glucose, galactose, glucuronic acid, mannose, and arabinose with a molar ratio of 97.39:1.64:0.29:0.22:0.21. Moreover, extraction and purification methods may affect the monosaccharide composition and proportion of CYPs. When the enzymatic method was used to assist the extraction, Liu et al. [41] found that the neutral fraction CYP1 isolated from the Chinese yam is composed of arabinose, galactose, glucose, and mannose in a molar ratio of 0.01:0.08:0.32:0.59. However, two acidic fractions, namely, CYP2 and CYP3, are composed of arabinose, galactose, glucose, mannose, and galacturonic acid with molar ratios of 0.08:0.50:0.15:0.18:0.09 and 0.04:0.09:0.69:0.13:0.04, respectively. Zhou et al. [27] found that the monosaccharides in the Chinese yam bulbil polysaccharide fraction PYB-1 are mannose, rhamnose, glucose, and galactose with a molar ratio of 2.19:0.88:1.45:0.58. However, the monosaccharides of the fraction PYB-2 are mannose, rhamnose, glucose, and xylose with a molar ratio of 0.61:0.34:0.39:0.16. Many scholars found that CYPs contain uronic acids [28,39,44]. However, these acids were not detected in the present work. Finally, the fermentation process of *S. cerevisiae* CICC 32883 may selectively alter certain monosaccharides (such as mannose) because of the preference and priority of microbial utilization [30,45]. Monosaccharides may affect the bioactivity and application of CYP-NF and CYP-SC by influencing their electrification and functional groups, which are discussed in the following work.

### 3.3. Molecular Weight Analysis

As shown in Table 1, the weight-average molecular weight and the number-average molecular weight of CYP-NF were 124.774 and 18.963 kDa, respectively, and its polydispersity was 6.58. After fermentation by *S. cerevisiae* CICC 32883, the weight-average molecular weight, number-average molecular weight, and polydispersity of CYP-SC reduced to 20.384 kDa, 6.033 kDa, and 3.379, respectively. This finding indicated that CYP-SC had a lower molecular weight and higher homogeneity than CYP-NF. On the one hand, extraction and purification methods might affect the molecular weight of yam polysaccharides. Liu et al. [41] found that the molecular weights of polysaccharide fractions extracted from the Chinese yam with enzymatic assistance differ. CYP1 contained five molecular weight fractions: 2.94 × 10^5^, 1.30 × 10^5^, 4.97 × 10^4^, 6.08 × 10^3^, and 1.38 × 10^3^ Da. CYP1 contained two molecular weight fractions: 3.43 × 10^5^ and 4.63 × 10^4^ Da. However, CYP3 only contained one fraction: 9.26 × 10^3^ Da. On the other hand, enzymes and/or organic acids secreted by yeast may reduce yam polysaccharide molecular weight by breaking glycosidic bonds [30]. Wang et al. [32] found that the molecular weight of *L. barbarum* polysaccharide decreases from 5.304 × 10^6^ g/mol to 2.231 × 10^4^ g/mol after being fermented by rice wine yeast. Chen et al. [33] suggested that the molecular weight of *D. officinale* polysaccharide decreases from 1268.21 ± 10.23 kDa to 25.74 ± 0.21 kDa with *S. cerevisiae* FBKL2.8022 fermentation. Pei et al. [34] also found that *S. cerevisiae* W5 fermentation reduces the molecular weight of blue honeysuckle polysaccharide from 105.60 kDa to 82.10 kDa. In the present work, the extraction conditions of yam polysaccharides were the same. Only *S. cerevisiae* CICC 32883 was used for fermentation pretreatment. This approach may be the main reason for the molecular weight reduction in CYP-SC. Molecular weights may affect the morphology, size, spatial configuration, absorption, and utilization rates of CYP-NF and CYP-SC, thereby influencing their bioactivity and applications.

### 3.4. FT-IR

**Figure 1:**
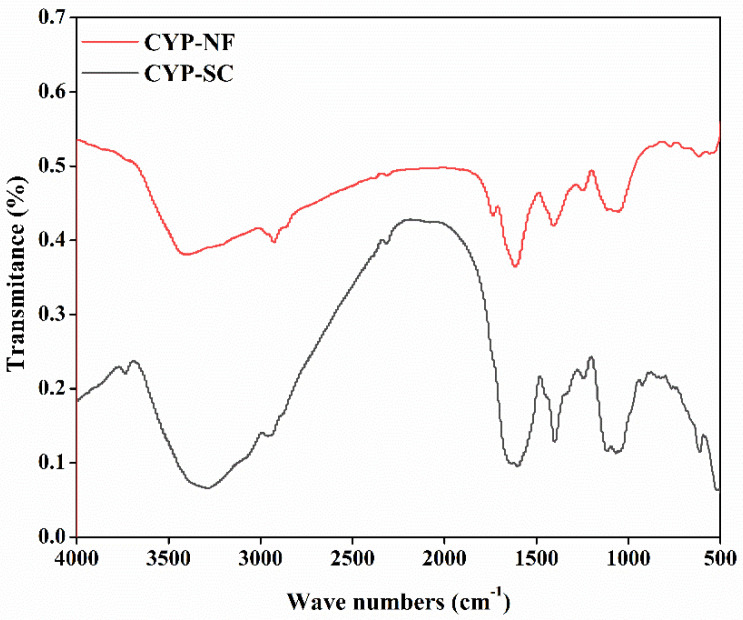
FT-IR spectra of CYP-NF and CYP-SC.

Functional groups in CYP-NF and CYP-SC, analyzed by FT-IR, may affect their bioactivity and application. As shown in Figure 1, the peaks between 3400 and 3200 cm^−1^ may relate to the intermolecular H-bridge of OH groups and OH stretching in CYP-NF and CYP-SC, and the peaks between 3000 and 2900 cm^−1^ may relate to the CH_2_ antisymmetric stretch in CYP-NF and CYP-SC; they were characteristic absorption peaks of polysaccharides [19]. The peaks between 1800 and 1700 cm^−1^ may relate to COOH groups or C=O stretching from acetyl in CYP-NF and CYP-SC, and the peaks between 1600 and 1400 cm^−1^ may relate to the CH_2_ symmetric ring stretching or CH_2_ scissor vibration in CYP-NF and CYP-SC [37]. The peaks between 1400 and 1100 cm^−1^ may relate to OH in-plane deformation, C-O-C antisymmetric stretching, and C-O stretching in CYP-NF and CYP-SC, and the peaks between 900 and 500 cm^−1^ may relate to C-anomeric group stretching and pyran ring stretching in CYP-NF and CYP-SC [38]. CYP-NF and CYP-SC had similar FT-IR spectra but different peak heights (Figure 1), indicating that CYP-NF and CYP-SC had the same functional group types but different amounts. This finding also indicated that *S. cerevisiae* CICC 32883 fermentation was inclined to break the main chain linkages of yam polysaccharide and reduce its molecular weight. The changes in structural features (including monosaccharide, molecular weight, and functional group) between CYP-NF and CYP-SC induced by *S. cerevisiae* CICC 32883 fermentation may provide a green and simple method for researchers to investigate the structure–activity relationship of polysaccharides.

### 3.5. In Vitro Antioxidant Activity Analysis

**Figure 2:**
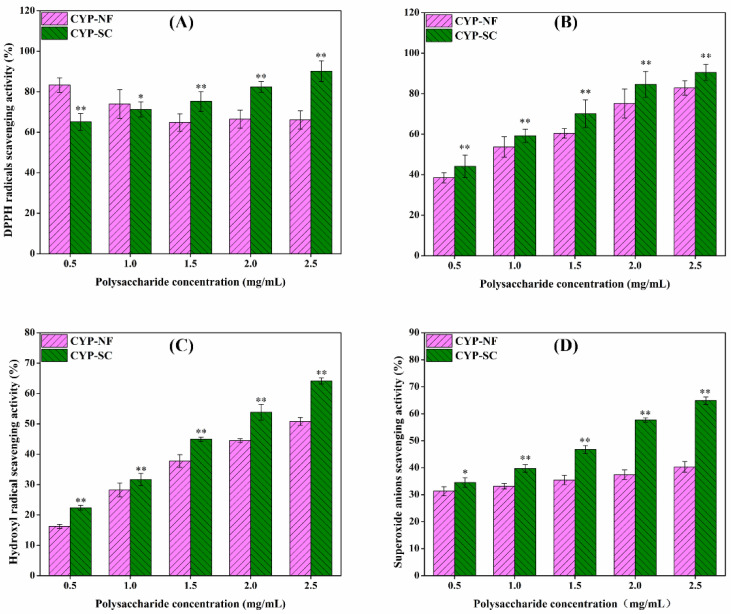
Antioxidant activity of CYP-NF and CYP-SC against DPPH (**A**), ABTS (**B**), hydroxyl (**C**), and superoxide (**D**) radicals. Significant difference as compared to CYP-NF was designated as * *p* < 0.05, ** *p* < 0.01.

Excessive ROS, which leads to inflammation, heart disease, tumors, cancer, and other diseases, is the primary cause of human aging and diseases [1,3]. As shown in Figure 2A,D, the scavenging effect of CYP-NF against DPPH radicals decreased slightly with increasing concentrations from 0.5 mg/mL to 2.5 mg/mL. Moreover, the scavenging superoxide radical effect slightly increased with increasing concentration. Figure 2B,C show that the scavenging effects of CYP-NF against ABTS and hydroxyl radicals increased with increasing polysaccharide concentration. However, the scavenging effects of CYP-SC against DPPH, ABTS, hydroxyl, and superoxide radicals showed a positive correlation with polysaccharide concentration (Figure 2). At a concentration of 2.5 mg/mL, the scavenging effects of CYP-NF against DPPH, ABTS, hydroxyl, and superoxide radicals were 66.11% ± 4.48%, 82.88% ± 3.58%, 50.80% ± 1.26%, and 40.33% ± 1.99%, respectively, but those for CYP-SC increased to 90.22% ± 5.10%, 90.54% ± 3.99%, 64.13% ± 0.97%, and 64.88% ± 1.41%. The changes in the chemical composition, monosaccharide, molecular weight (Table 1), and functional group (Figure 1) affected by *S. cerevisiae* CICC 32883 may influence the bioactivities of CYP-SC, and antioxidant activity is one of them. Many researchers obtained results similar to those obtained in the present work, indicating that microbial fermentation improved the antioxidant activity of polysaccharides. Shao et al. [31] found that *S. boulardii* fermentation improves the antioxidant activities of CYP against DPPH and ABTS radicals, and total reducing power is determined by reducing its molecular weight and changing the morphological structure. Moreover, Yang et al. [46] and Yu et al. [47] found that *Lactobacillus casei* and *L. plantarum* FM 17 fermentation increase the antioxidant activity of *Polygonatum kingianum* and jackfruit polysaccharides against free radicals, possibly because of the molecular weight reduction and change in monosaccharide proportion.

### 3.6. Antioxidant Activity Analysis on the HepG2 Cell Model

**Figure 3:**
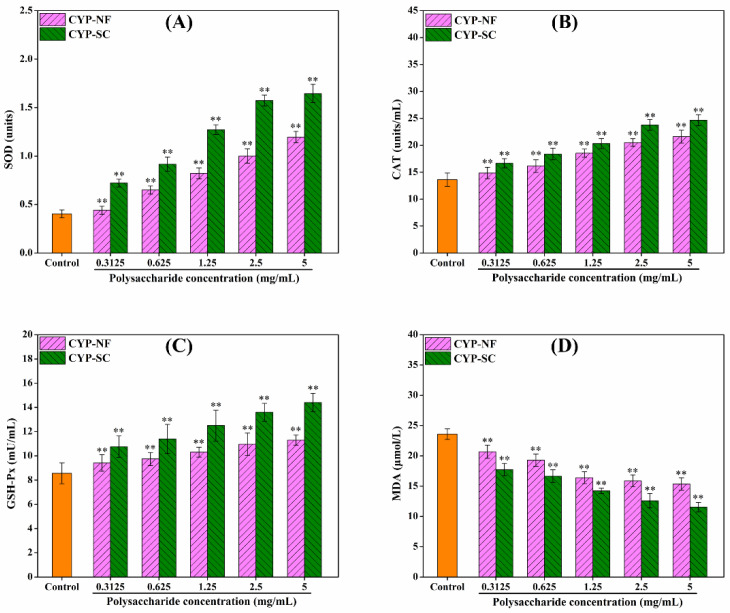
Effect of CYP-NF and CYP-SC on SOD (**A**), CAT (**B**), GSH-Px (**C**), and MDA (**D**) activities in HepG2 cells. Significant differences as compared to control group were designated as ** *p* < 0.01.

**Figure 4:**
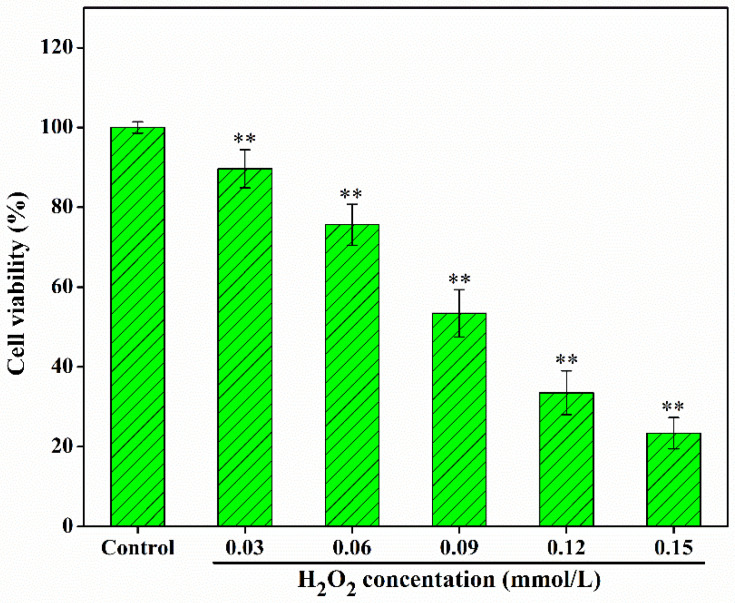
Effect of H_2_O_2_ concentration on the viability of HepG2 cells. Significant differences as compared to 100% cell activity were designated as, ** *p* < 0.01.

**Figure 5:**
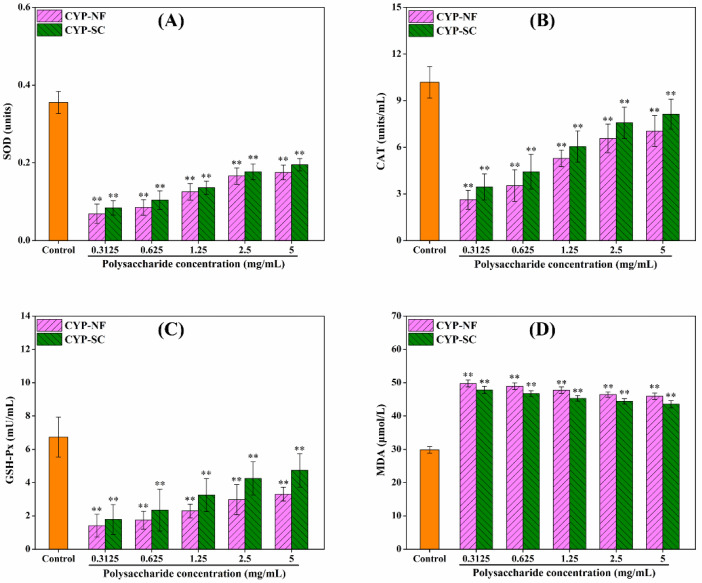
Effect of CYP-NF and CYP-SC on H_2_O_2_-damaged prevention of HepG2 cells. SOD (**A**), CAT (**B**), GSH-Px (**C**), and MDA (**D**) activities. Significant differences as compared to control group were designated as ** *p* < 0.01.

**Figure 6:**
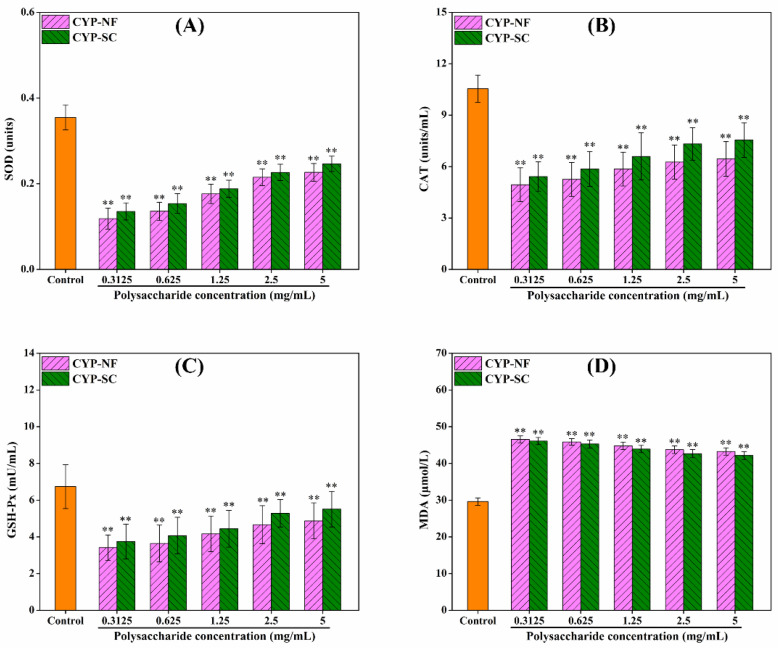
Effect of CYP-NF and CYP-SC on H_2_O_2_-damaged alleviation of HepG2 cells. SOD (**A**), CAT (**B**), GSH-Px (**C**), and MDA (**D**) activities. Significant differences as compared to control group were designated as, ** *p* < 0.01.

**Figure 7:**
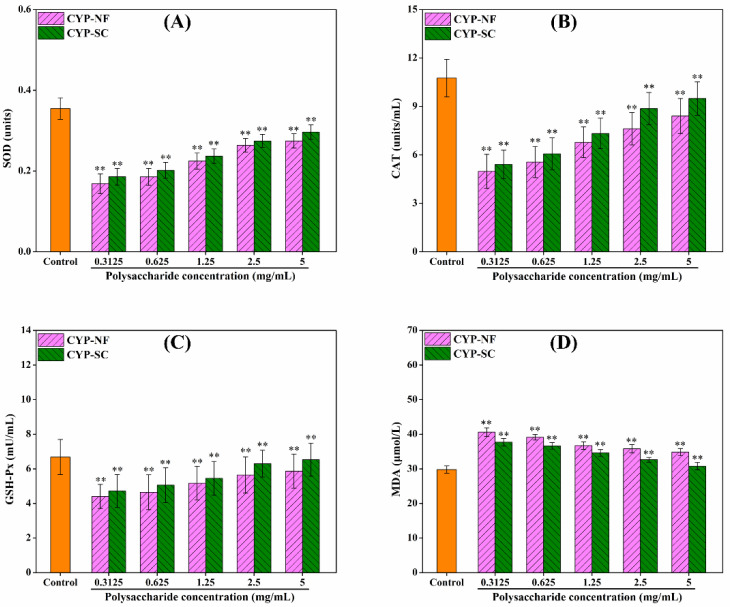
Effect of CYP-NF and CYP-SC on H_2_O_2_-damaged repairment of HepG2 cells. SOD (**A**), CAT (**B**), GSH-Px (**C**), and MDA (**D**) activities. Significant differences as compared to control group were designated as ** *p* < 0.01.

Improving oxidative capacity and antioxidant activity can reduce the damage of ROS to the human body. As shown in Figure 3, CYP-NF and CYP-SC improved the oxidative capacity of HepG2 cells and showed a positive correlation with polysaccharide concentration. Different concentrations of H_2_O_2_ were used to construct the H_2_O_2_-damaged HepG2 cell model. Figure 4 shows that the cell viability was 52.13% ± 2.59% when H_2_O_2_ was 0.09 mmol/L. This finding can be used in the following work. After the HepG2 cells were treated by CYP-NF (or CYP-SC) with different concentrations for 24 h, continuous treatment with 0.09 mmol/L H_2_O_2_ for 6 h resulted in weak damage on HepG2 cells, as shown in Figure 5. Moreover, reducing ROS-induced damage and repairing oxidative damage caused by ROS is important. As shown in Figure 6, CYP-NF and CYP-SC could alleviate the damage induced by H_2_O_2_ in HepG2 cells when HepG2 cells were treated with combined 0.09 mmol/L H_2_O_2_ and different concentrations of CYP-NF (or CYP-SC). Furthermore, Figure 7 also shows that CYP-NF and CYP-SC excellently repaired the oxidative damage induced by H_2_O_2_ in HepG2 cells. Therefore, CYP-NF and CYP-SC showed a full range of antioxidant capacity and oxidative protection activities, and CYP-SC had better effects than CYP-NF, possibly because of the reduced protein and polyphenol contents in CYP-SC, change in monosaccharide proportion, and decrease in its molecular weight effect by *S. cerevisiae* CICC 32883 fermentation [30]. The above results suggested that CYP-SC may be potentially used as an antioxidant ingredient in food and medicinal fields.

The antioxidant protection effect of CYPs has been widely recognized by scholars. Shen et al. [48] found that CYP protects against H_2_O_2_-induced oxidative damage in IEC-6 cells by restraining MAPK signaling pathway activation. Li et al. [44] found that CYP improves SOD activity and reduces ROS and MDA contents in H_2_O_2_-damaged IEC-6 cells. Zhou et al. [27] investigated the antifatigue effects of CYP and found that CYP increases SOD and GSH-Px contents and decreases MDA content in exhaustive swimming mice. Moreover, the increasing antioxidant protection effect of plant polysaccharides by yeast fermentation is attracting increasing attention. You et al. [49] found that *S. cerevisiae* CMCC17452–fermented *Panax notoginseng* root polysaccharides decrease the ROS and MDA contents and reverse the expressions of CAT, GSH-Px, and SOD in H_2_O_2_-induced human dermal fibroblast. Furthermore, the combined fermentation with yeast is a good strategy to improve the antioxidant protection effect of plant polysaccharides. Chen et al. [35] found that *S. cerevisiae* CGMCC 2.119 and *Bacillus subtilis* CGMCC 1.892 fermentation considerably improve the antioxidant protection effect of wheat bran polysaccharides to reduce ROS production in an AAPH-induced zebrafish model.

## 4. Conclusions

Microbial fermentation could increase the extractability of plant polysaccharides and could modify their structure and bioactivity. In this study, *S. cerevisiae* CICC 32883 fermentation increased the carbohydrate content of CYP from 71.03% ± 2.75% (CYP-NF) to 84.86% ± 2.92% (CYP-SC), and the molecular weight was reduced from 124.774 kDa (CYP-NF) to 20.384 kDa (CYP-SC). However, the monosaccharide composition and proportion of CYP-SC were similar to those of CYP-NF. On the one hand, CYP-SC had higher scavenging activities against DPPH, ABTS, hydroxyl, and superoxide radicals than CYP-NF. On the other hand, CYP-SC showed better effects in protecting, alleviating, and repairing the H_2_O_2_ damage on HepG2 cells than CYP-NF. Regretfully, the fermentation kinetics of *S. cerevisiae* CICC 32883 and extraction conditions of CYPs were not optimized. Meanwhile, it is hoped that metabolic engineering technologies can be used to improve modifying efficiency and realize industrial production in future work. On the whole, the present work will provide a method for improving the antioxidant activity of dietary plant polysaccharides and the production of functional ingredients and foods.

## Figures and Tables

**Table 1:** Chemical composition and structural characteristics of Chinese yam polysaccharides before and after fermentation by *Saccharomyces cerevisiae* CICC 32883.

Chemical Composition	CYP-NF	CYP-SC
Carbohydrate contents (%)	71.03 ± 2.75	84.86 ± 2.92 **
Protein content (%)	8.24 ± 0.19	7.12 ± 0.19 **
Polyphenol content (%)	0.26 ± 0.07	0.13 ± 0.04 **
**Monosaccharide composition (μg/mL)**		
Rhamnose	0.493	0.1543 **
Arabinose	0.6695	0.1635 **
Galactose	0.9738	0.5759 **
Glucose	0.7655	0.5286 **
Mannose	12.4365	3.2247 **
**Molecular weight (kDa)**		
Weight-average molecular weight (M_w_)	124.774	20.384 **
Number-average molecular weight (M_n_)	18.963	6.033 **
Polydispersity (M_w_/M_n_)	6.58	3.379 **

Data are expressed as means ± SD (n = 3). Significant difference as compared to CYP-NF was designated as ** *p* < 0.01.

## Data Availability

The original data presented in the study can be requested from the corresponding author.
